# 643. The Complete Story: Packaged Testing and the Gaps in Sexually Transmitted Infection Screening

**DOI:** 10.1093/ofid/ofaf695.207

**Published:** 2026-01-11

**Authors:** Tri Pham, Nick Cardoza, Germysha Little, Andrew Atkinson, Benjamin Cooper, Hilary Reno

**Affiliations:** Washington University in St. Louis School of Medicine, St. Louis, MO; Washington University in St. Louis, St. Louis, Missouri; Washington University in St. Louis, St. Louis, Missouri; Washington University School of Medicine, St. Louis, Missouri; Washington University in St. Louis, St. Louis, Missouri; Washington University in St. Louis, St. Louis, Missouri

## Abstract

**Background:**

Concurrent, or “packaged,” testing for human immunodeficiency virus (HIV) and syphilis is recommended for individuals undergoing gonorrhea and chlamydia (GC/CT) screening due to elevated risk of co-infections. Incomplete testing can result in continued transmission and delayed treatment of sexually transmitted infections (STIs). We aimed to assess the prevalence of and factors associated with packaged STI testing in a large hospital and clinic system.

**Figure 1.**
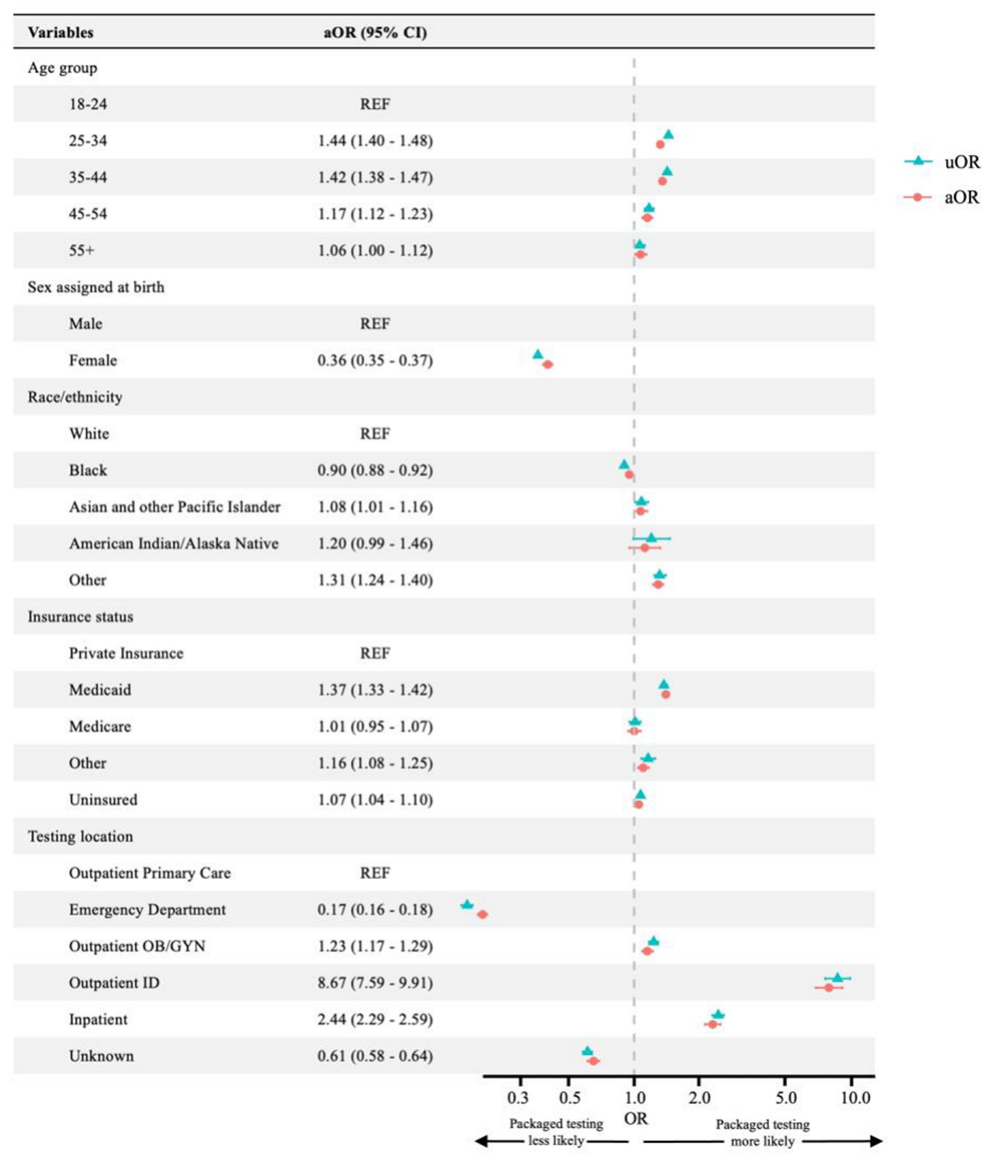
Forest plot of uni- and multivariable logistic regression of factors associated with receipt of packaged testing This figure represents the results of the uni- and multivariable logistic regression models estimated using generalized estimating equations fitted to receipt of packaged testing as the dependent variable. Packaged testing refers to the concurrent testing for HIV, syphilis, and GC/CT. The blue represents the results of the univariable model, while the red represents the results of the multivariable model. Abbreviations used: aOR, adjusted odds ratio; CI, confidence intervals; GC/CT, gonorrhea/chlamydia; HIV, human immunodeficiency virus; ID, infectious diseases; SE, standard error; OB/GYN, obstetrics and gynecology; uOR, unadjusted odds ratio.

**Figure 2.**
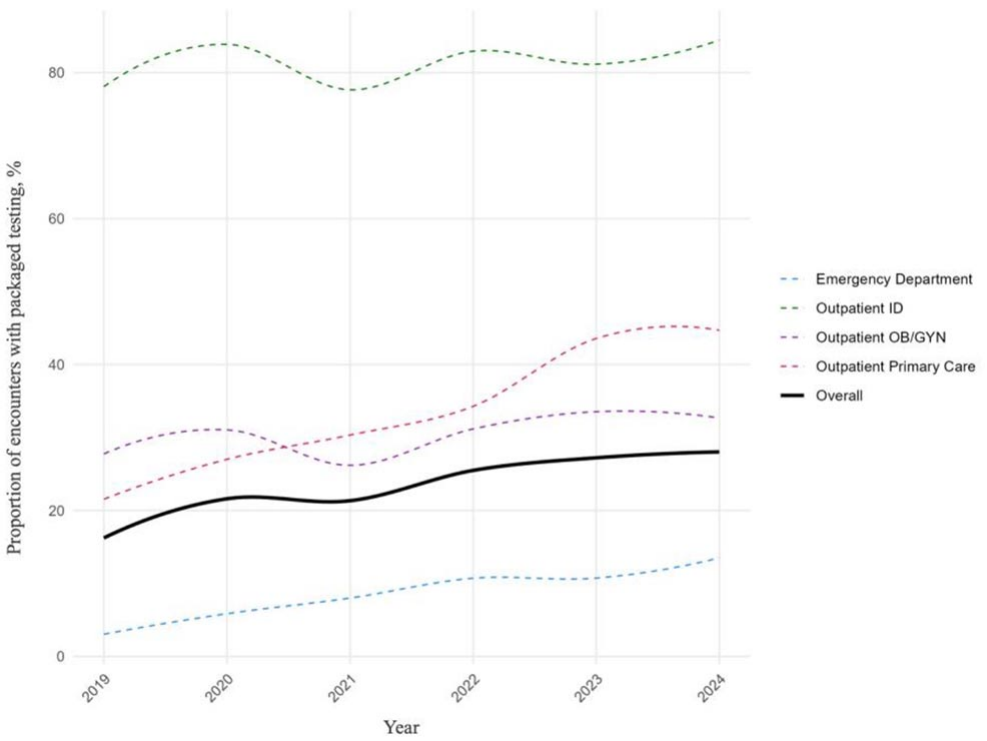
Trends in packaged testing across healthcare settings

This figure depicts the trends in packaged testing across different healthcare settings. This represents the number of encounters with packaged testing in proportion to the total number of encounters within the specific location group. The solid black line represents the overall trend in our cohort. The green dashed line represents ID clinic encounters, the purple dashed line represents OB/GYN clinic encounters, the red dashed line represents primary care clinic encounters, and the blue dashed line represents emergency department encounters. Abbreviations used: ID, infectious disease; OB/GYN, obstetrics and gynecology.

**Methods:**

We analyzed adult patient encounters from January 2019 through December 2024 with concomitant GC/CT testing at a large Midwestern academic hospital system. Encounters after a patient’s first positive HIV test were excluded. We defined packaged testing as concurrent testing for HIV, syphilis, and GC/CT. Uni- and multivariable logistic regression models estimated using generalized estimating equations were fitted with receipt of packaged testing as the dependent variable, using sandwich-type standard errors to account for potential correlation from multiple encounters per individual.

**Table 1.**
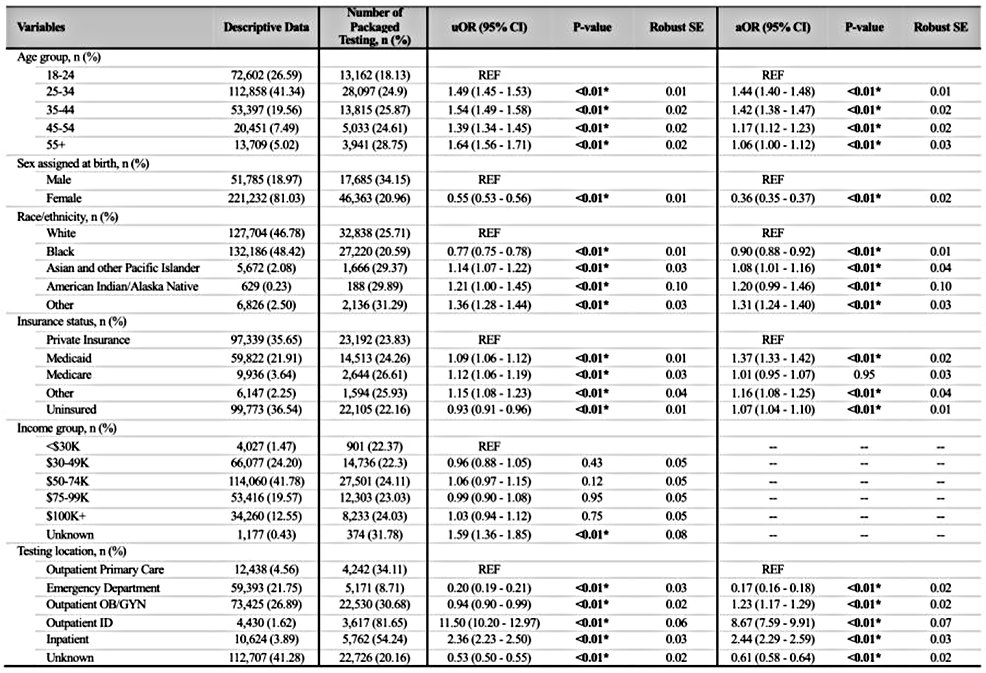
Demographics and regression analyses of factors associated with receipt of packaged testing This table presents demographics, rate of packaged testing across categories, and results from both univariable and multivariable logistic regression analyses estimated using generalized estimating equations. The "Other" category for race/ethnicity includes individuals identifying as multiracial or Hispanic. The "Other" category for insurance status refers to workers' compensation, organizational billing, or other non-disclosed insurance types. Median household incomes were estimated based on residential zip codes, which were linked to data from the United States Census Bureau. *p-value < 0.05. Abbreviations used: aOR, adjusted odds ratio; CI, confidence intervals; IQR, interquartile range; ID, infectious diseases; SE, standard error; OB/GYN, obstetrics and gynecology; uOR, unadjusted odds ratio.

**Results:**

We identified 150,874 individuals with 273,017 encounters, of which 64,048 (23.5%) received packaged testing. Those aged ≥ 25 had higher odds of receiving packaged testing compared to younger individuals. Female sex (adjusted odds ratio [aOR] 0.35, 95% confidence interval [CI] 0.34-0.37) and Black race (aOR 0.89, 95% CI 0.87-0.91) were associated with lower odds compared to male sex and White patients, respectively. Medicaid coverage was associated with the highest odds of receiving packaged testing (aOR 1.37, 95% CI 1.33-1.42; reference: private insurance). Encounters at infectious disease clinics (aOR 5.6, 95% CI 4.99-6.27) and inpatient settings (aOR 2.43, 95% CI 2.28-2.59) had higher odds, whereas emergency department encounters had lowest odds (aOR 0.16, 95% CI 0.16-0.17), all relative to outpatient primary care. Rates of packaged testing increased over successive calendar years.

**Conclusion:**

In a large Midwestern hospital system, packaged testing prevalence remains low, with younger individuals, females, and Black patients less likely to receive complete testing. Disparities across settings underscore opportunities for protocol-driven interventions and provider education to improve screening practices.

**Disclosures:**

Hilary Reno, MD, PhD, Hologic Inc: Grant/Research Support

